# SpikeID: Rapid and unbiased identification of SARS-CoV-2 variants by spike sequencing

**DOI:** 10.1016/j.jcv.2025.105845

**Published:** 2025-07-28

**Authors:** Keith Farrugia, Zain Khalil, Adriana van de Guchte, Bremy Alburquerque, Daniel Floda, PSP Study Group, Komal Srivastava, Luz H. Patiño, Juan David Ramirez, Alberto E. Paniz-Mondolfi, Emilia Mia Sordillo, Viviana Simon, Ana S. Gonzalez-Reiche, Harm van Bakel

**Affiliations:** a Department of Genetics and Genomic Sciences, Icahn School of Medicine at Mount Sinai, New York, NY 10029, USA; b Department of Microbiology, Icahn School of Medicine at Mount Sinai, New York, NY 10029, USA; c Center for Vaccine Research and Pandemic Preparedness (C-VaRPP), Icahn School of Medicine at Mount Sinai, New York, NY 10029, USA; d Department of Pathology, Molecular, and Cell-Based Medicine, Icahn School of Medicine at Mount Sinai, New York, NY 10029, USA; e School of Sciences and Engineering, Universidad del Rosario, Bogotá, Colombia; f Division of Infectious Diseases, Department of Medicine, Icahn School of Medicine at Mount Sinai, New York, NY 10029, USA; g Icahn Genomics Institute, Icahn School of Medicine at Mount Sinai, New York, NY 10029, USA; h Department of Artificial Intelligence and Human Health, Icahn School of Medicine at Mount Sinai, New York, NY 10029, USA; i The Global Health Emerging Pathogens Institute, Icahn School of Medicine at Mount Sinai, New York, NY 10029, USA

**Keywords:** Long-read sequencing, Virus evolution, Genotyping, Molecular surveillance, SARS-CoV-2, Virus variants, Spike

## Abstract

**Background:**

Severe acute respiratory syndrome coronavirus 2 (SARS-CoV-2) variants of concern (VOCs) are characterized by distinct mutations in the S1 domain of the viral spike protein. This domain encompasses the N-terminal domain, the receptor-binding domain, and part of the cleavage site region. While mutations in other genomic regions of SARS-CoV-2 can impact VOC potential, the S1 domain holds particular importance for identifying variants and assessing antigenic evolution and immune escape potential.

**Methods:**

We describe a rapid high-throughput sequencing-based assay, SpikeID, for the unbiased detection and identification of SARS-CoV-2 variants based on spike S1 amplicon sequencing. We benchmarked the SpikeID assay against Illumina whole-genome sequencing across 622 clinical biospecimens, representing lineages that circulated globally from October 2021 to January 2024.

**Results:**

SpikeID unambiguously detected 100 % of WHO-designated VOCs and identified PANGO lineages circulating at ≥1 % prevalence in the New York City (NYC) area with 93 % accuracy in comparison to whole-genome sequencing. This reduction in accuracy was largely due to PANGO lineages that are only distinguishable by mutations outside the S1 domain.

**Conclusions:**

We demonstrate the utility and scalability of the SpikeID assay during the emergence and subsequent surge of Omicron and Omicron-derived lineages in New York City, and show that our approach enables cost-effective, reliable, and near-real-time detection of emerging lineages.

## 1. Introduction

Severe acute respiratory syndrome coronavirus 2 (SARS-CoV-2) variants of concern (VOCs) continue to emerge. These viral variants carry mutations that enhance transmissibility, increase virulence, and/or enable evasion of existing immunity [1]. Many, including currently circulating Omicron-derived variants, are also partially or fully resistant to therapeutic or prophylactic monoclonal antibody treatments [2,3]. Rapid identification of VOCs is important for optimizing antiviral treatment allocation, strengthening health system preparedness, and supporting effective public health responses. Considering the ongoing decline of resources available for global SARS-CoV-2 genomic surveillance [4–6], implementation of cost-effective, rapid sequencing methods that capture viral diversity is critical to maintaining effective monitoring to ensure timely detection of newly emerging highly mutated variants.

SARS-CoV-2 genomic diversity is categorized into lineage designations based on whole-genome sequencing data [7], with the PANGO nomenclature established as the predominant system for global variant tracking. Notably, many of these lineages can be accurately identified using only the spike protein sequence [8]. The S1 domain of the spike protein is the most dominant immunogenic antigen and one of the most rapidly evolving regions in the SARS-CoV-2 genome [9]. Several sequencing-based methods are available that target the spike region to identify VOCs, using short-read Illumina, Oxford Nanopore, or Sanger sequencing [10–12]. Most existing protocols rely on tiled amplification strategies requiring multiple primer sets and separate amplification reactions, along with reference-based assembly approaches for consensus sequence generation. In addition, these methods have been evaluated only *in silico* using genome sequences deposited in public repositories, validated on limited numbers of clinical samples, or deployed for only short periods of time across small numbers of variants [13,14].

To facilitate rapid typing of SARS-CoV-2 variants, we developed the ’SpikeID’ assay, a targeted single-amplicon sequencing approach focusing on key S1 antigenic regions of the spike protein that harbor many of the signature mutations defining VOC lineages. An integrated protocol combines an experimental workflow with an analysis pipeline, achieving a 24-h turnaround time in a 96-well plate format using only benchtop equipment. We highlight the utility and scalability of the SpikeID assay through profiling of 3358 nasopharyngeal and saliva specimens collected during multiple SARS-CoV-2 surges in NYC. Integration of SpikeID as part of our SARS-CoV-2 surveillance algorithm enabled the early detection of the Omicron variant in November 2021 and the JN.1 lineage in October 2023.

## 2. Methods

### 2.1. Molecular SARS-CoV-2 diagnostics and sample selection

SARS-CoV-2 molecular diagnostics were conducted at the Molecular Microbiology Laboratories of the Mount Sinai Hospital (MSH) Clinical Laboratory in New York City using nucleic acid amplification tests (NAAT) on nasopharyngeal (NP), anterior nares (AN) swabs, and saliva specimens, as previously described [15]. The study protocol was approved by the Icahn School of Medicine Program for the Protection of Human Subjects (ISMMS PPHS) Institutional Review Board (STUDY-13-00981). This study covers the period between March 1st, 2020, and January 8th, 2024.

### 2.2. Illumina whole-genome amplification and sequencing

The SARS-CoV-2 whole-genome sequencing (WGS) data used in this study were generated by the Mount Sinai Pathogen Surveillance Program (MS-PSP) from samples collected between February 29, 2020, and January 8, 2024, using previously described methods [15,16].

### 2.3. SpikeID sequencing of the S1 domain

**Fig. 1.**
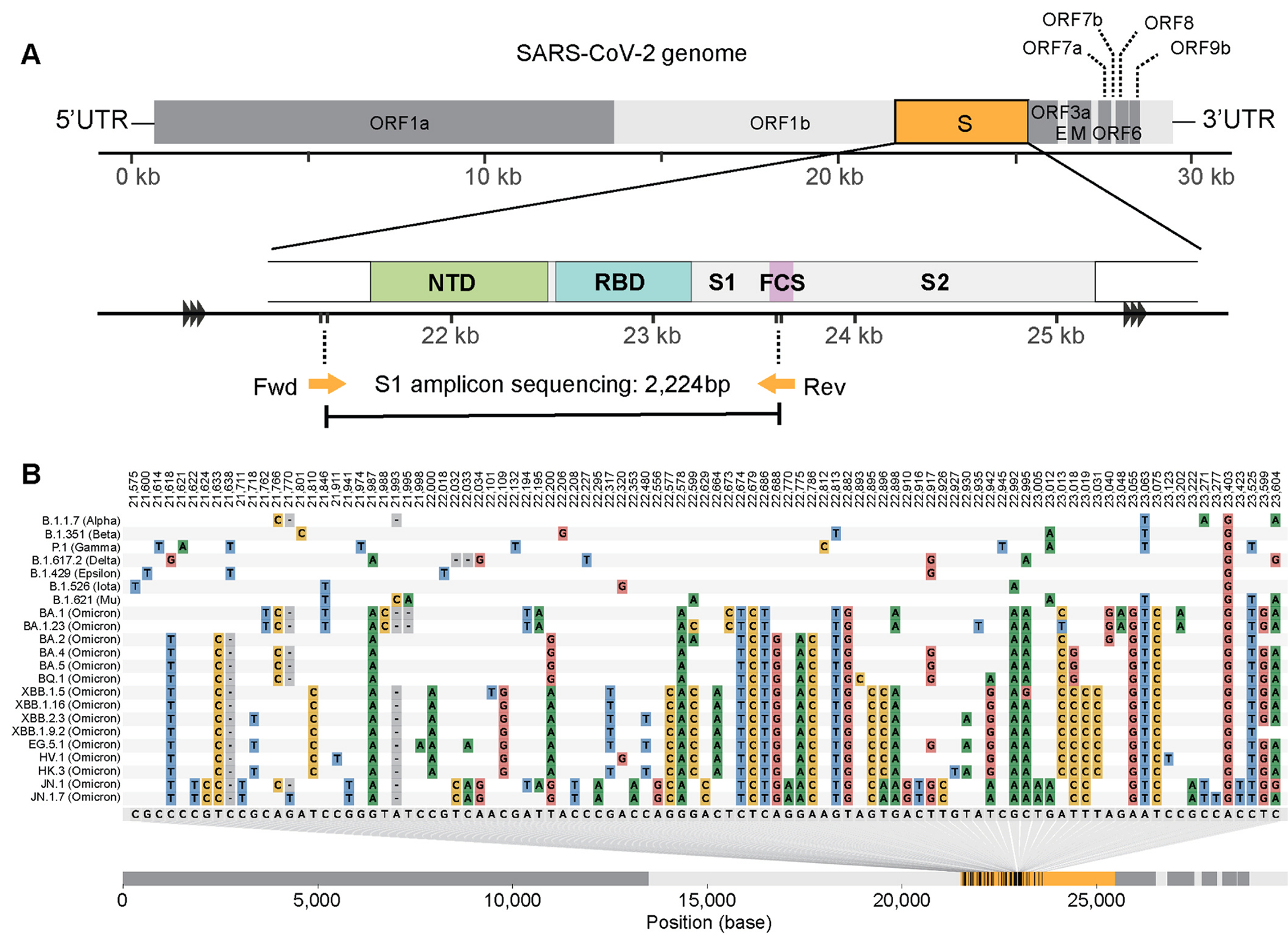
SpikeID assay for the identification of SARS-CoV-2 variants. (A) Amplification strategy of the Spike S1 domain for SpikeID. The binding region for the forward and reverse primers are indicated by the orange arrows. Primer sequences are flanked by ONT barcode, spacer, and adapter sequences. (B) Distinct mutational profiles for major variants of concern and lineages of interest based on the S1 region targeted by SpikeID. The figure was produced with representative samples as observed in New York City by the MS-PSP program, and using Snipit (https://github.com/aineniamh/snipit) with manual aesthetic modifications in Adobe Illustrator^®^.

A step-by-step protocol is provided in the Supplementary material. The SpikeID assay forward (5′-ACAAATCCAATTCAGTTGTCTTCCTATTC-3′) and reverse (5′-TGACTAGCTACACTACGTGCCC-3′) primers were designed to target flanking regions of the S1 domain of the SARS-CoV-2 spike gene that were highly conserved (99.9 % (Fig. 1). We assessed the primer performance throughout the study period by aligning the primer sequences to prototypical sequences of novel variants as they emerged. The latest sequence conservation analysis with 15 million SARS-CoV-2 genomes from GISAID [6] [downloaded on January 6, 2024], showed that these regions remained conserved >99.9 % and with minimal changes between 2020 and 2024 (Supplementary Table 1). These primers were barcoded with Oxford Nanopore Technologies (ONT) native barcode index sequences (Oxford Nanopore, cat. #SQK-NBD110.96) to allow multiplexing. Additional flanking sequences compatible with ONT’s analysis software were added.

### 2.4. SpikeID data analysis

We developed a custom Snakemake [17,18] workflow to assemble and genotype spike S1 amplicon sequences (Supplementary Figure 2). Basecalling and demultiplexing were performed on FAST5/POD5 files using Guppy (v6.4.6 + ae70e8f), in combination with minimap2 [19] (v2.24-r1122). The basecalling models were applied based on their respective sequencing flow cells (Supplementary Table 2).

The SpikeID analysis workflow supports two distinct methods for generating consensus spike sequences: *de novo* assembly using AmpliconSorter [20] or reference-guided assembly using the ARTIC pipeline [21]. For *de novo* assembly with AmpliconSorter, raw reads are processed with Cutadapt (v.4.5) [22], retaining only reads longer than 2 kb. The filtered FASTQ files are then subsampled to a maximum of 10,000 reads per sample, sorted using AmpliconSorter (v.2023-06-19), and polished with Medaka (v.1.8.0) [23]. The final consensus S1 sequences are reoriented to the sense strand of the Wuhan-Hu-1 reference genome (GenBank NC_045512.2). For the reference-based approach, the raw demultiplexed FASTQ reads are aligned using the ARTIC pipeline [21] (v.1.2.4). SpikeID primer coordinates are subsequently used to trim the primer binding regions, followed by consensus sequence polishing using Medaka (v1.8.0) against NC_045512.2 using the models specified in Supplementary Table 2. For both approaches, a minimum coverage of 100 mapped reads is required for a sample to pass quality control (QC).

While both ARTIC and AmpliconSorter produce similar results, the reference-free approach with AmpliconSorter is the default in the pipeline and was used to generate the SpikeID consensus sequences used in this study. An advantage of this approach is that the consensus is derived from reads that match the predetermined amplicon size without the need for a specific reference.

### 2.5. SARS-CoV-2 genotyping

Consensus sequences obtained from the WGS and SpikeID assays were genotyped using Nextclade v.3.2.1 [24], and Pango [25] lineage assignments were extracted from the ‘Nextclade_pango’ column. For samples collected after April 16, 2022, genotyping was done against a modified NC_045512.2 that incorporates BA.2 signature variants, as provided by Nextclade [24]. To compare lineage calls between Illumina WGS data and Nanopore SpikeID domain-specific data, the Illumina-sequenced genomes were trimmed to match the SpikeID amplicon coordinates and analyzed for lineage assignment using the same method.

### 2.6. PANGO lineage consolidation

To focus on the most prevalent lineages while reducing the impact of low-prevalence lineages, we developed a lineage consolidation algorithm (Supplementary Figure 3). This algorithm groups lineages with low-prevalence and represents them by their closest parental lineage. Specifically, a minimum count threshold *c_P_* is defined as *c_P_* = *nP*, where *n* is the total number of sequences in the dataset, and *P* is the desired prevalence threshold. PANGO lineages that do not meet the count threshold *c_P_* are systematically collapsed. For any lineage *i* where *c_i_* < *c_P_*, the lineage *i* is replaced by its immediate parent in an iterative manner. This process continues until the cumulative counts of the parent lineage meet or exceed the predefined *c_P_* threshold. To ensure that VOCs were retained in our analyses regardless of their frequency, WHO-designated lineages such as alpha (B.1.1.7), beta (B.1.351), gamma (P.1), delta (B.1.617.2), eta (B.1.525), epsilon (B.1.427/429), iota (B.1.526), kappa (B.1.617.1), lambda (C.37), mu (B.1.621), and omicron (B.1.1.529) were preserved.

### 2.7. Cost and time assessment

To assess the cost-effectiveness of SpikeID, we estimated the total and hands-on time, as well as the per-sample cost, for SpikeID and WGS sample preparation (Supplementary Tables 3 and 4). These estimates were based on a 96-sample run, a commonly used batch size in laboratories performing routine sequencing workflows.

## 3. Results

### 3.1. SpikeID matches whole-genome sequencing in sensitivity for SARS-CoV-2 surveillance

**Fig. 2.**
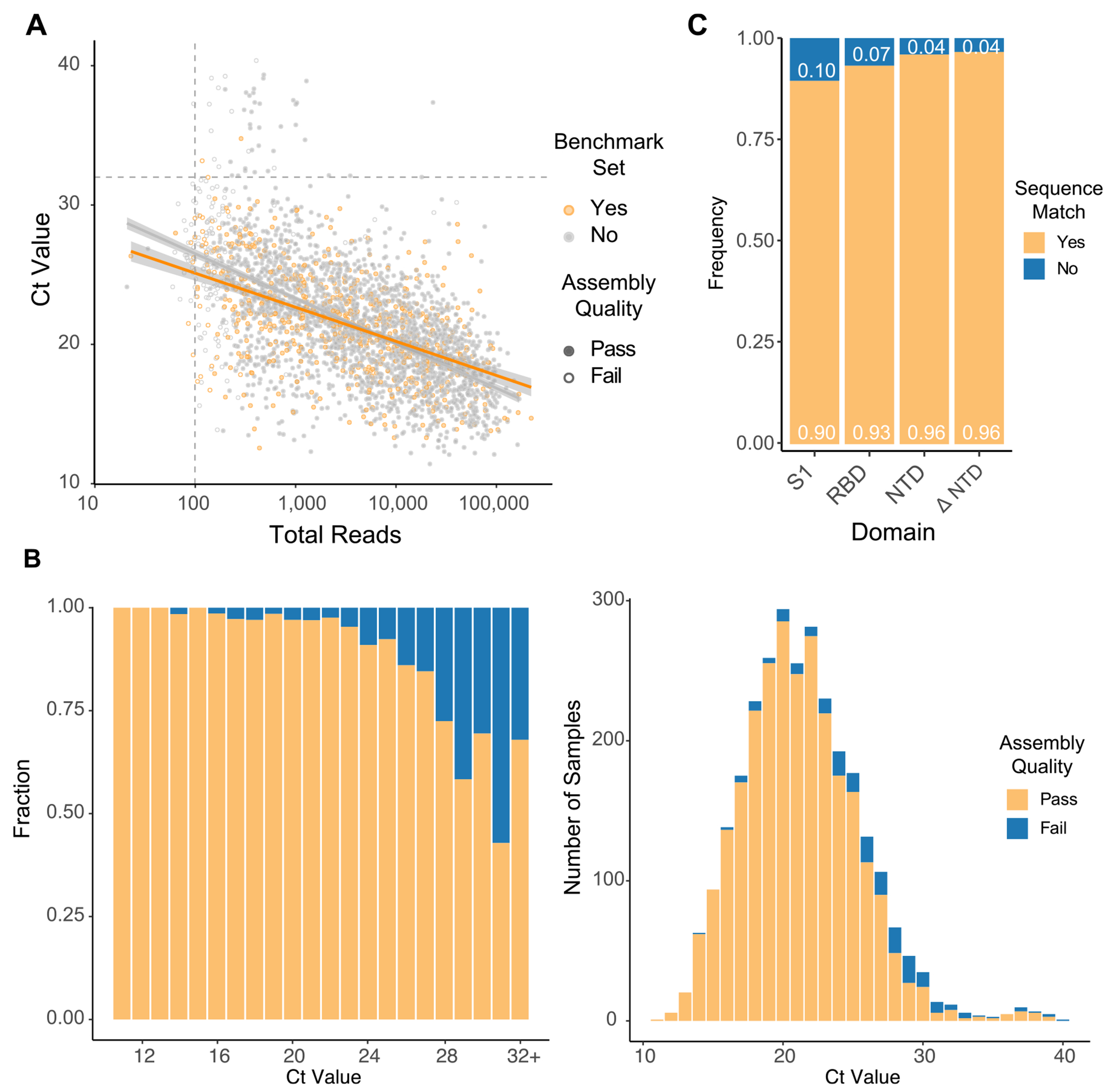
SpikeID sensitivity and accuracy. (A) Diagnostic Ct values versus read counts for 2963 samples distributed across 68 sequencing runs. The samples that were benchmarked against WGS are shown in orange. Samples that passed or failed assembly QC are shown in filled circles or empty circles respectively. The linear regression between Ct value ~ Total reads is shown for each set of samples. (B) Frequency of samples by assembly quality, grouped by diagnostic Ct value unit. (C) Fraction of sequences from SpikeID with identical matches to Illumina WGS data displayed for the different Spike regions for 622 specimens sequenced on both platforms. S1 domain; RBD, receptor-binding domain; NTD, N-terminal domain; Δ_NTD_, deletions in the N-terminal domain.

We initially deployed the SpikeID assay in 2021 to supplement ongoing SARS-CoV-2 whole-genome sequencing efforts during COVID-19 surges to enhance our ability to detect novel, emerging VOCs (Fig. 1). Between October 3rd, 2021, and January 8th, 2024, we assayed 4020 clinical specimens obtained from SARS-CoV-2-positive patients seeking care within the Mount Sinai Health System (MSHS) with SpikeID. Following quality control to verify the presence of valid primer sequences on both ends, we generated full-length S1 amplicon read sequences for 3646 clinical samples (90.7 %) across 68 assay runs, with each run accommodating up to 95 samples (Fig. 2).

To assess the sensitivity of the SpikeID assay, we examined the relationship between the Cycle threshold (Ct) values from diagnostic testing of the clinical specimens with available Ct data (n = 2963) and the number of S1 amplicon reads detected by SpikeID. As expected, given that lower Ct values correspond to higher viral loads, we observed a negative correlation between Ct values and S1 amplicon read counts (Fig. 2A). The fraction of samples that passed read-level quality control decreased as Ct values increased, suggesting that assay dropouts were predominantly caused by low viral loads in clinical samples (Fig. 2B). Overall, most samples with a diagnostic Ct ≤ 32 produced at least 100 sequencing reads, which is consistent with the pass rate we and others have observed for whole-genome sequencing in the same Ct range [26, 27]. A small subset (n = 32; 5 %) did not reach the minimum required sequencing depth of 100 reads, despite having Ct values below 32 and yielding complete genomes on the Illumina platform. These samples showed no SpikeID primer mismatches based on their Illumina genomes, suggesting that the low sequencing depth was likely due to sample quality or preparation issues. For all but three of these samples, the SARS-CoV-2 lineages identified were consistent with those from the Illumina genomes when the sequencing depth threshold was lowered to 50 reads.

At the more conservative threshold of 100 reads, 3358 samples (92 %) successfully passed SpikeID assembly quality control. Of these, 622 (18.5 %) also had complete genome sequences available generated on the Illumina platform. This provided a benchmarking dataset to evaluate SpikeID performance that included all 10 WHO-designated VOCs of the Delta and Omicron lineages circulating between the Spi- keID sample collection period (October 3rd, 2021–January 8, 2024).

We assessed the quality of S1 amplicon sequences generated on the Nanopore platform using the 622 samples benchmark set with paired SpikeID and Illumina whole-genome sequences. Overall, 90 % of specimens yielded identical S1 sequences, with concordance rates of 93 % for the receptor-binding domain (RBD) and 96 % for the N-terminal domain (NTD), specifically. Nucleotide-level discrepancies between the methods were primarily due to consensus sequence errors at the 5′ or 3′ ends of the S1 amplicon compared to WGS, or indels in low complexity regions, a known limitation of the Nanopore platform (Fig. 2C) [28,29].

### 3.2. SpikeID identifies VOCs and their prevalent sub-lineages with high accuracy

**Fig. 3.**
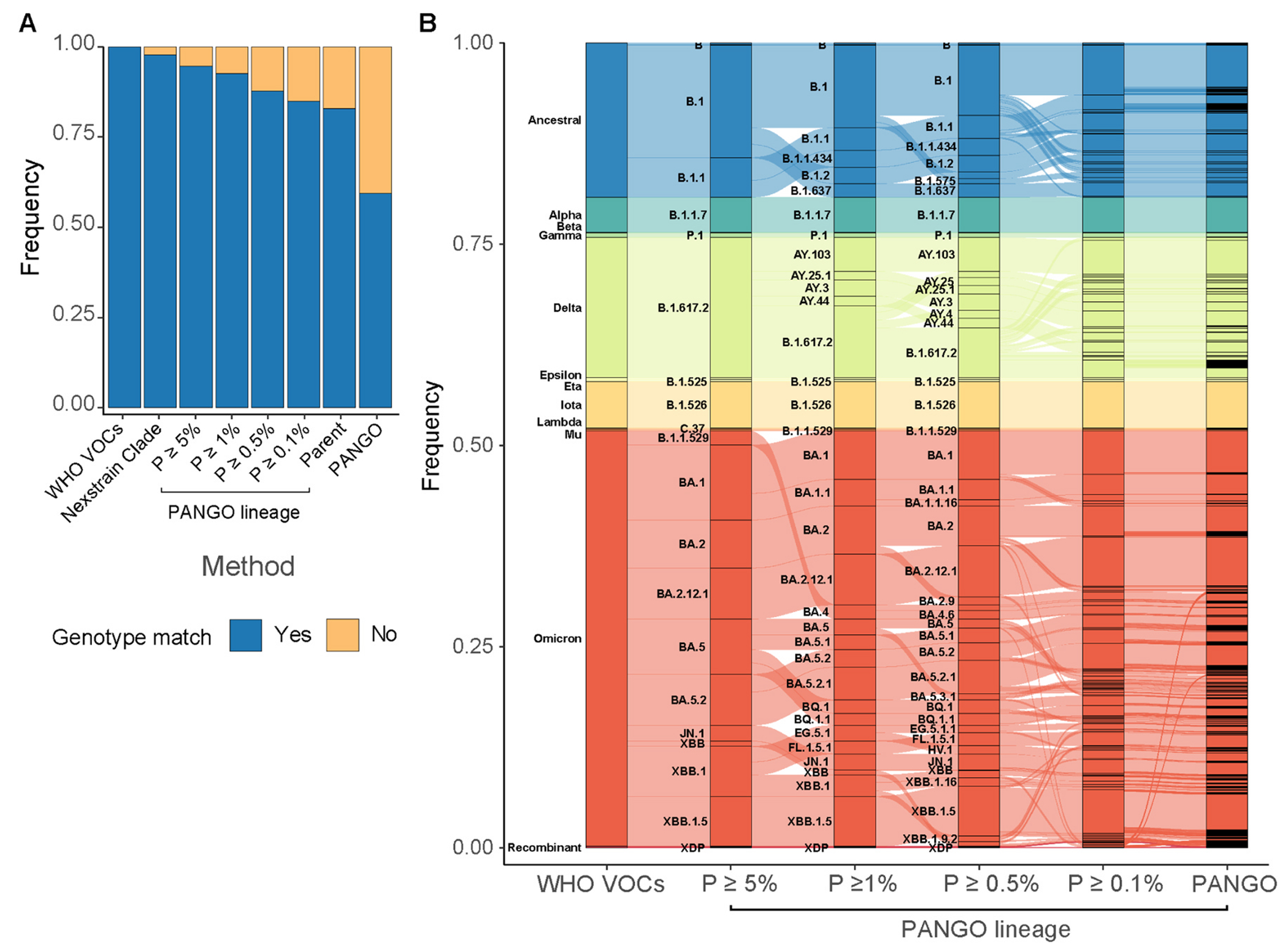
Accuracy of SARS-CoV-2 variant and lineage calling for the SpikeID assay. (A) Benchmarking of the accuracy of the SpikeID assay to WGS paired data at different genotype classifications including the WHO-designated VOCs, Nextstrain Clades (20C, 21I-L, 22A-F, 23A-F, 24A, and recombinant), and collapsed lineages based on the S1 region at prevalence thresholds of 0.1 %, 0.5 %, 1 %, and 5 %, respectively. Each of the SpikeID genotype calls were matched to the consolidated lineages. The accuracy of the SpikeID assay was then defined as the ratio of the number of samples with a matching lineage over the total number of samples in the benchmarking set. (B) Lineages collapsed at different prevalence thresholds (*P*) to assess the SpikeID assay genotyping accuracy. PANGO lineages are grouped and colored by ancestral or WHO-designated VOCs and shown in the first column for reference. The top lineages are listed for *P* ≤ 0.1 %, 0.5 %, 1 % and 5 %.

We used the benchmark set to evaluate SpikeID’s lineage classification accuracy across phylogenetic resolutions with Nextclade. To account for the marked genetic divergence of Omicron from earlier SARS-CoV-2 lineages, we used two reference datasets: Wuhan-Hu-1 for sequences before April 16, 2022, and Wuhan-Hu-1 with BA.2 signature variants thereafter. Accuracy was calculated for the 622 clinical biospecimens that passed both Illumina WGS and SpikeID assembly QC, and using the Illumina lineage call as the reference. SpikeID achieved 100 % accuracy in identifying variant-level designations, including all WHO- designated VOCs and recombinant lineages. It also achieved 98 % accuracy in classifying specimens according to the main clade hierarchy defined by Nextstrain, encompassing 19 distinct Nextstrain clades represented in our benchmark dataset (Fig. 3A). Although we used three nanopore flow cell chemistries (R9.4.1, R10.3, and R10.4) to generate the benchmark set, we did not compare their lineage-calling accuracy, as R10 flow cells were used for fewer samples and only during the circulation of later SARS-CoV-2 variants (Table S2), which could have confounded the results.

To evaluate the accuracy of SpikeID genotype and lineage calls below the VOC level, we developed an aggregation algorithm that consolidates low-frequency sub-lineages into their more common parent lineages. This approach was applied for lineage prevalence thresholds ranging from 0.1 % to 5 % in our comprehensive SARS-CoV-2 genomic surveillance dataset of 14,210 MSHS specimens from the NYC metropolitan area analyzed between March 2020 and January 2024. The benchmark set of 622 specimens with paired genotyping data contained 170 distinct PANGO lineages as identified by WGS, of which 95 were also identified by SpikeID based on S1 sequence alone (59 % concordance). When sublineages were consolidated into their more prevalent parent lineages (Fig. 3B), concordance rates between SpikeID and WGS lineage calls improved progressively at prevalence thresholds of 0.1 %, 0.5 %, 1 %, and 5 %, reaching 85 %, 88 %, 93 % and 95 %, respectively (Fig. 3A). Thus, although SpikeID does not capture the full lineage diversity represented in WGS data, it reliably identified the 46 out of the 51 more common parent lineages (≥1 % prevalence) circulating in the NYC metropolitan area over the three-year study period, with *>*90 % accuracy.

### 3.3. SpikeID effectively captures SARS-CoV-2 lineage dynamics compared to WGS

**Fig. 4.**
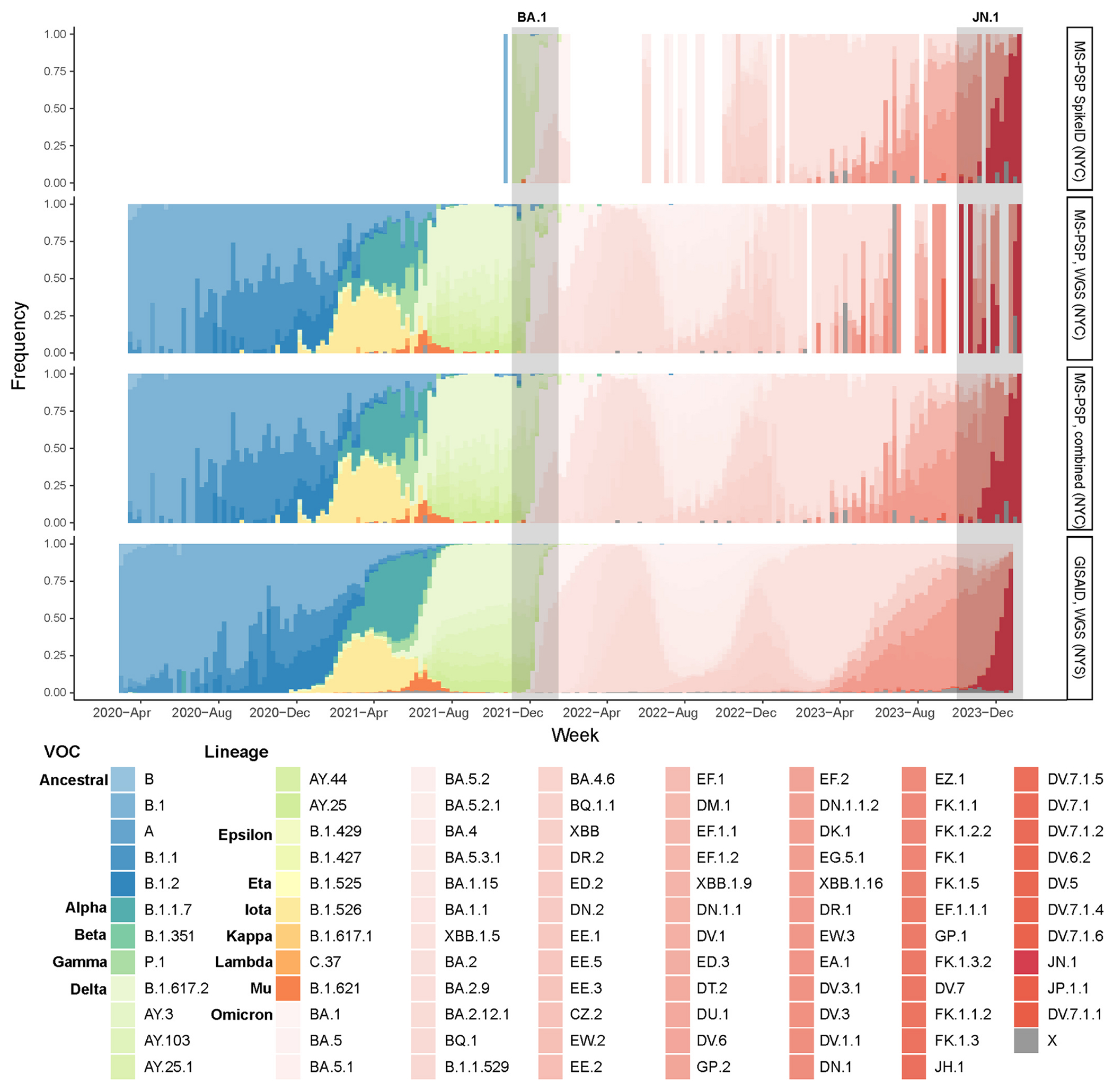
Genomic surveillance of SARS-CoV-2 in New York City with SpikeID. SARS-CoV-2 relative lineage distribution over time between March 2020 and March 2024. The lineages detected by SpikeID and benchmarked against WGS paired data are highlighted. For the remaining lineages, a 1% prevalence threshold was used for collapsing them to their closest parent lineage. The first and second panels show SpikeID results for 3438 samples and WGS results for 11,075 samples from MS-PSP surveillance in the NYC region, respectively. The third panel presents the combined results from both methods. The fourth panel shows the lineage distribution for publicly available data available in GISAID for 331,994 samples from NYS, after excluding the MS-PSP data deposited in GISAID. The shaded boxes highlight regions where SpikeID data detected rapid variant displacement in NYC during the introduction of Omicron BA.1 in November of 2021 and JN.1 in October of 2023.

To assess SpikeID’s effectiveness in tracking SARS-CoV-2 lineage dynamics, we compared SpikeID-profiled data with MS-PSP WGS surveillance data from the NYC metropolitan area and publicly available GISAID data from New York State (NYS), using the latter due to inconsistent county-level reporting. Lineages were consolidated at a 1 % prevalence threshold across all datasets. This allowed for the comparison of lineage distribution and diversity captured by SpikeID and WGS in NYC. As shown in Fig. 4, SpikeID accurately and timely captured the overall distribution of lineages with at least 1 % prevalence in the NYC area, including the emergence of Omicron and the subsequent displacement of Delta variants (shaded area toward the end of 2021). Using the SpikeID assay we identified cases of the Omicron (BA.1) variant as early as November 27, 2021, representing one of the earliest official reports of this VOC in NYS [30]. Subsequent sequencing efforts revealed the rapid dissemination of Omicron in NYC and NYS, with its prevalence increasing in the ensuing weeks. By the first week of December, a total of 48 (9.3 %) of the sequenced specimens were identified as Omicron BA.1 variant (B.1.1.529.1) using the SpikeID assay, matching state-wide data deposited in GISAID.

Throughout the Omicron period and up to the conclusion of this study in January 2024, SpikeID continued to provide a reliable representation of circulating variants in New York City. Notably, SpikeID also detected the appearance of the JN.1 variant as early as October 2023, further highlighting its robustness for monitoring emerging lineages with increasing prevalence in the health system. By January 2024, SpikeID effectively captured the near-complete displacement of XBB.1- derived lineages (second shaded area at the end of 2023).

Lastly, we estimated the time and costs required to process a 96-well sample plate using the SpikeID protocol, compared to our Illumina WGS protocol. While reagent costs may vary by region, our side-by-side comparison shows that the SpikeID assay requires less hands-on time and can be completed within a single working day, unlike the Illumina WGS assay. Additionally, the same number of samples can be screened and genotyped at approximately one-fifth of the reagent cost (Supplementary Tables 3 and 4).

## 4. Discussion

In this study, we demonstrate that the SpikeID assay matches WGS in sensitivity and can effectively monitor SARS-CoV-2 lineage dynamics over time. By profiling mutation patterns in the spike gene, SpikeID accurately detected major WHO-designated VOCs and their prevalent sub-lineages. Across 3629 high-quality clinical samples collected over two years, SpikeID exhibited strong concordance with WGS, particularly for specimens with lower Ct values (≤32). Consequently, we recommend preselection of samples with a Ct value equal to or less than 32 for this assay. Furthermore, benchmarking against WGS and GISAID data confirmed SpikeID’s robustness in tracking the emergence, displacement, and temporal dynamics of prevalent SARS-CoV-2 lineages, including the Omicron variant and the more recent lineages. These findings underscore SpikeID as a sensitive, cost-effective, and reliable alternative to WGS for timely variant monitoring in clinical and public health settings.

SpikeID has proved to be a robust and adaptable tool across multiple SARS-CoV-2 waves dominated by divergent variants. Initial primer optimization ensured the assay’s reliability against early lineages (e.g., B.1, Alpha, Beta, Gamma, Mu, and Iota). Our sequence conservation analysis confirmed that SpikeID targets are highly conserved, showing minimal variation in the primer binding sites across over 15 million global genomes (Supplementary Table 2). Nonetheless, we recommend periodically assessing the primer binding regions using contemporary genomes from public sequence repositories. This approach, particularly when complemented by a genotyping assay, such as a reflex test, or WGS of samples that do not yield SpikeID results even when their Ct values meet the recommended threshold, can help mitigate dropouts and support unbiased variant detection within surveillance areas. As of mid-2025, SpikeID remains effective in detecting emerging variants, including XFG, LF.7, NB.1.8.1, LP.8.1, among other lineages. We therefore expect it to remain an unbiased and effective tool for detecting future SARS-CoV-2 lineages for estimating the relative abundance of co-circulating variants in ongoing surveillance efforts.

Compared to WGS, SpikeID’s high-throughput capacity and expedited turnaround times make it well suited for integration into rapid response strategies. During COVID-19 surges, the deployment of SpikeID allowed us to double screening capacity and reduce turnaround times to less than two days. This is particularly important for the timely identification of spike protein mutations in the N-terminal domain (NTD) and receptor-binding domain (RBD), enabling swift updates to monoclonal antibody therapies and spike protein-based vaccines to address immune escape variants. Additionally, SpikeID can be a valuable tool for rapid genotyping during outbreak investigations and contact tracing, further supporting its role in proactive SARS-CoV-2 surveillance and response.

Beyond surge response, SpikeID offers a cost-effective, scalable solution for sustaining genomic surveillance as COVID-19 funding declines. Its use of affordable Nanopore sequencing makes it accessible for adoption in both high- and low-income settings, helping to close gaps in global genomic surveillance. By reducing costs while maintaining high accuracy for prevalent lineages, SpikeID enables sustained, real-time monitoring of SARS-CoV-2 evolution and variant emergence, ensuring essential surveillance infrastructure remains intact. As the virus continues to evolve, tools like SpikeID will be crucial for detecting emerging variants, informing public health responses, and safeguarding global preparedness against future outbreaks.

## Supplementary Material

1

2

## Data Availability

The SpikeID analysis pipeline is available at https://zenodo.org/records/15881340. Source data and code for generating the figures in this manuscript are available at https://github.com/BakelLab/manuscript_SpikeID. Nucleotide sequences are available in GenBank (Supplementary Table 5).

## Appendix A. Supporting information

Supplementary data associated with this article can be found in the online version at doi:10.1016/j.jcv.2025.105845.
