# Supplementary material for: SpikeID: Rapid and unbiased identification of SARS-CoV-2 variants by spike sequencing
Source: J Clin Virol. Author manuscript; Available in PMC 2026 Jun 12. (PMC13262703; doi:10.1016/j.jcv.2025.105845)
Supplement: 1 [file NIHMS2180501-supplement-1.pdf]

Supplementary material

SpikeID: Rapid and unbiased identification of SARS-CoV-2 variants by spike sequencing

Authors: Keith Farrugia[1*], Zain Khalil[1*], Adriana van de Guchte[1], Bremy Alburquerque[1], Daniel Floda[1], PSP Study Group**, Komal Srivastava[2,3], Luz H. Patiño[4,5], Juan David Ramirez[4,5], Alberto E. Paniz-Mondolfi[4], Emilia Mia Sordillo[4], Viviana Simon[2,3,4,6,9], Ana S. Gonzalez-Reiche[1+†], Harm van Bakel[1,2,7,8+†].

1. Department of Genetics and Genomic Sciences, Icahn School of Medicine at Mount Sinai, New York, NY, 10029, USA.
2. Department of Microbiology, Icahn School of Medicine at Mount Sinai, New York, NY, 10029, USA.
3. Center for Vaccine Research and Pandemic Preparedness (C-VaRPP), Icahn School of Medicine at Mount Sinai, New York, NY, 10029, USA.
4. Department of Pathology, Molecular, and Cell-Based Medicine, Icahn School of Medicine at Mount Sinai, New York, NY, 10029, USA.
5. Facultad de Ciencias Naturales, Universidad del Rosario, Bogotá, Colombia
6. Division of Infectious Diseases, Department of Medicine, Icahn School of Medicine at Mount Sinai, New York, NY, 10029, USA.
7. Icahn Genomics Institute, Icahn School of Medicine at Mount Sinai, New York, NY, 10029, USA.
8. Department of Artificial Intelligence and Human Health, Icahn School of Medicine at Mount Sinai, New York, NY, 10029, USA.
9. The Global Health Emerging Pathogens Institute, Icahn School of Medicine at Mount Sinai, New York, NY, 10029, USA.

* Equal contribution. † Co-Senior authors. ** PSP Study Group team members are listed in the Acknowledgments.

+Correspondence: anasilvia.gonzalez-reiche@mssm.edu, harm.vanbakel@mssm.edu

This document contains:

- Supplementary SpikeID protocol.
- Supplementary tables 1 to 4.
- Supplementary figures 1 to 3.

SpikeID assay protocol

A) Molecular SARS-CoV-2 Diagnostics and sample selection

This protocol was developed and validated using residual respiratory specimens from patients seeking care at the Mount Sinai Health System (MSHS). The specimens included NP and AN swabs and some saliva samples. All specimens were collected, accessioned and banked by the Mount Sinai Pathogen Surveillance Program (MS-PSP) after completion of the standard of care clinical diagnostic process.

B) RNA extraction

Viral RNA was extracted from the banked clinical specimens using the Chemagic^{TM} Viral DNA/RNA 300 Kit H96 (cat.# IVD-1033-S) on the Chemagic^{TM} Instrument according to the manufacturers' instructions.

C) Complementary DNA (cDNA) synthesis

cDNA synthesis was conducted using the ProtoScript II kit (NEB, cat. # E6560L) according to the manufacturer's protocol. Briefly, 3.5 µl of sample RNA were combined with 3.5 µL nuclease-free water and 1 µL of random hexamers and incubated at 65°C for 5 minutes to denature the RNA. Subsequently, a master mix containing 10 µL of ProtoScript II buffer and 2 µL of ProtoScript II reverse transcriptase was added to each sample. The reaction was carried out under the following thermal conditions: 25°C for 5 minutes for primer annealing, 48°C for 40 minutes for cDNA synthesis, and 80°C for 5 minutes for enzyme inactivation.

D) Illumina whole genome sequencing

Samples with a SARS-CoV-2 NAAT cycle threshold (Ct) value ≤ 32 were amplified with two sets of custom primers to produce overlapping 1.5 and 2kb amplicons spanning the SARS-CoV-2 genome. Paired-end libraries were then prepared from pooled amplicons with the Nextera XT DNA Sample Preparation Kit (Illumina cat. #FC-131-1096) and sequenced on a MiSeq instrument with the Reagents kit v2 (500-cycles, cat. #MS-102-2003). Genomes were assembled using a custom reference-guided pipeline, vRAPID[1, 2] using Wuhan-Hu-1 genome (GenBank NC_045512.2) as the reference.

E) SpikeID Primer sequences for the S1 domain

The primers are composed of nucleotide sequences that target regions flanking the S1 domain:

Forward sequence (*SpikeID_Fwd*) : 5'-ACAAATCCAATTCAGTTGTCTTCCTATTC-3'

Reverse sequence (*SpikeID_Rev*): 5'-TGACTAGCTACACTACGTGCCC-3'

In addition, they contain 96 different sequences matching the ONT native barcode index sequences (Oxford Nanopore, cat. #SQK-NBD110.96) to allow multiplexing. Additional flanking sequences were added to ensure the amplicons are compatible with ONT's analysis software. A 5' phosphorylation is added to ensure the final amplicon is ready for ONT adapter ligation. The final primer design is as follows:

- <u>Forward primer</u>: 5'-/Pho/ AAGGTTAA - barcode - CAGCACCT- *SpikeID_Fwd*- 3'
- <u>Reverse primer</u>: 5'-/Pho/ GGTGCTG - barcode - TTAACCTTAGCAAT- *SpikeID_Rev*-3'

F) SpikeID Reverse transcriptase and PCR amplification

For each sample, a SpikeID PCR mixture was prepared containing 12.5 μL nuclease-free water, 5 μL Platinum™ II Taq Hot-Start DNA Buffer (Invitrogen, cat. #14966025), 0.5 μL dNTPs (NEB,

cat. #N0447L), 1 µL forward primer (10 nM), 1 µL reverse primer (10 nM), 0.25 µL Platinum™ II Taq Hot-Start DNA Polymerase, and 5 µL cDNA. Amplification was conducted under the following conditions: initial denaturation at 98°C for 30 seconds, followed by 32 cycles of 98°C for 15 seconds and 63°C for 3 minutes, with a final extension step at 65°C for 10 minutes.

G) SpikeID library preparation and sequencing

Amplified DNA (20 µL per sample) was purified using 0.45X SPRI beads (AMXPure, Beckman Coulter, cat. #A63822). The beads were agitated for 5 minutes at 1,000 rpm, washed twice with 125 µL of 70% ethanol, and eluted in 27.5 µL nuclease-free water with 5 minutes of agitation at 1,000 rpm. Barcoded spike S1 amplicons from up to 96 samples were then pooled, and 25 µL of clarified supernatant was used for adapter ligation.

For Nanopore adapter ligation, 50 ng of pooled amplicon DNA were diluted to 30 µL in LoBind tubes (Eppendorf, cat. #022431021). The mixture included 5 µL of Ligation Adapter (Oxford Nanopore, SQK-LSK109 or SQK-LSK114), 10 µL of NEBNext Quick Ligation Reaction Buffer (5X), and 5 µL of Quick T4 DNA Ligase (New England Biolabs, cat. #M2200L). The reaction was incubated at room temperature for 30 minutes. Post-ligation, 20 µL of SPRI beads were added and incubated on a Hula Mixer for 10 minutes. Beads were washed twice with 125 µL of short fragment buffer and resuspended in 15 µL of elution buffer. The eluate was transferred to clean LoBind tubes.

For sequencing, a total of 30 fmol of DNA, calculated based on an amplicon size of 2,000 bp, were loaded onto a pre-primed R9 (FLO-MIN106D) or R10 (FLO-MIN114) flow cell in a Mk1B or Mk1C MinION instrument (Oxford Nanopore Technologies). Sequencing runs were conducted for 6–12 hours, achieving a minimum yield of 1 million reads per run (**Supplementary Figure 1**).

Supplementary Table 1. Conservation analysis for SpikeID primers across SARS-CoV-2 sequences from GISAID. The analysis included 15,396,879 global sequences for the forward primer, and 15,665,319 for reverse primer after removing sequences with ambiguous bases "N" across the primer region**.**

Primer	Genome Position	Consensus Base	% Most Conserved	Majority Variant	% Majority Variant
Spikeid_Fwd	21357	A	99.9951	G	0.0026
	21358	C	99.9903	T	0.0069
	21359	A	99.9964	G	0.0011
	21360	A	99.9969	G	0.0008
	21361	A	99.9914	G	0.0052
	21362	T	99.9617	C	0.0351
	21363	C	99.8820	T	0.1107
	21364	C	99.9180	T	0.0750
	21365	A	99.9815	G	0.0120
	21366	A	99.9840	T	0.0071
	21367	T	99.9933	C	0.0034
	21368	T	99.9616	C	0.0224
	21369	C	99.9828	G	0.0115
	21370	A	99.9882	G	0.0069
	21371	G	99.2885	T	0.6989
	21372	T	99.9962	C	0.0010
	21373	T	99.9970	G	0.0007
	21374	G	99.9882	A	0.0086
	21375	T	99.9959	G	0.0016
	21376	C	99.9970	A	0.0006
	21377	T	99.9907	C	0.0060
	21378	T	99.9942	C	0.0022
	21379	C	99.9906	T	0.0068
	21380	C	99.9844	T	0.0093
	21381	T	99.9955	G	0.0020
	21382	A	99.9977	G	0.0003
	21383	T	99.9925	C	0.0055
	21384	T	99.9959	C	0.0021
	21385	C	99.9954	T	0.0023

Primer	Genome Position	Consensus Base	% Most Conserved	Majority Variant	% Majority Variant
SpikeID_Rev	23609	G	99.9718	T	0.0146
	23610	G	99.9043	T	0.0593
	23611	G	99.9716	T	0.0129
	23612	C	99.9374	T	0.0579
	23613	A	99.9914	G	0.0033
	23614	C	99.9970	T	0.0003
	23615	G	99.9930	T	0.0038
	23616	T	99.9964	C	0.0007
	23617	A	99.9966	G	0.0006
	23618	G	99.9967	A	0.0005
	23619	T	99.9948	C	0.0023
	23620	G	99.9686	A	0.0163
	23621	T	99.9963	C	0.0012
	23622	A	99.9831	G	0.0117
	23623	G	99.9687	A	0.0149
	23624	C	99.7435	T	0.2475
	23625	T	99.9935	C	0.0037
	23626	A	99.9918	C	0.0033
	23627	G	99.9589	T	0.0322
	23628	T	99.9885	C	0.0048
	23629	C	99.9957	A	0.0007
	23630	A	99.9912	G	0.0039

Supplementary Table 2. Oxford Nanopore Flowcells and their corresponding basecalling and consensus polishing models that were used in this study.

Flowcell	Basecalling Model	Medaka Model	Number of Runs	Number of Samples (Benchmark set)
R.9.4.1	dna_r9.4.1_450bps_hac.cfg	r941_min_high_g360	45	2,604 (689)
R.10.3	dna_r10.3_450bps_hac.cfg	r10_min_high_g340	8	342 (26)
R.10.4	dna_r10.4.1_e8.2_400bps_hac.cfg	r1041_e82_400bps_hac_g632	16	1,074 (25)

Supplementary Table 3. Total and hands-on time for the SpikeID and Illumina based Whole Genome Sequencing assays [1,3].

Assay	Step	Description	Total Time (h)	Hands On time (h)
SpikeID assay				
	1	Reverse Transcription	1.00	0.25
	2	Polymerase Chain Reaction	2.42	0.17
	3	Pooling and SPRI clean up	0.65	0.25
	4	Qubit quantification	0.17	0.17
	5	Adapter Ligation	0.75	0.08
	6	Final cleanup and Qubit quantification	0.83	0.28
	7	Flowcell Loading	0.25	0.17
		Total	6.07	1.37
Illumina whole-genome sequencing				
	1	Reverse Transcription	1.00	0.25
	2	Polymerase Chain Reaction	3.72	0.50
	3	SPRI clean up	0.88	0.42
	4	QC Gel electroforesis and Quantification	0.62	0.37
	6	Sample dilutuions	1.00	1.00
	7	Tagmentation	0.32	0.17
	8	Nextera XT PCR	0.83	0.08
	9	Post PCR clean Up	0.72	0.17
	10	Normalization	1.28	0.42
	11	Final Pooling	0.08	0.08
	12	Flowcell Loading	2.75	2.75
		Total	13.20	6.20
SpikeID assay time / Illumina WGS assay time			0.5	0.2

Supplementary Table 4. Approximate cost per sample for SpikeID and Illumina-based whole genome sequencing [1,3] assays. The estimated cost is based on a sequencing run of 96 samples. Prices are approximate based on list prices applicable to the Icahn School of Medicine at Mount Sinai as of June 2025. Prices may change by region.

Step	SpikeID	WGS
Reverse Transcriptase	$3.56	$5.33
PCR	$2.18	$5.73
Primers	$0.04	$0.04
SPRI beads	$0.26	$0.46
Library preparation	$2.69	$41.88
QC (quantification)	$0.27	$0.67
Flowcell	$6.25	$18.00
General consumables	$0.17	$0.84
Total	$15.42	$72.95
SpikeID cost / Illumina WGS cost		0.21

References

1. Gonzalez-Reiche AS, Alshammary H, Schaefer S, et al. Sequential intrahost evolution and onward transmission of SARS-CoV-2 variants. Nat Commun **2023**; 14(1): 3235.
2. Khalil ZS, Mitch Gonzalez-Reiche, Ana S. Obla, Ajay van Bakel, Harm. vRAPID: Virus reference-based assembly pipeline and identification. Zenodo, **2023**.

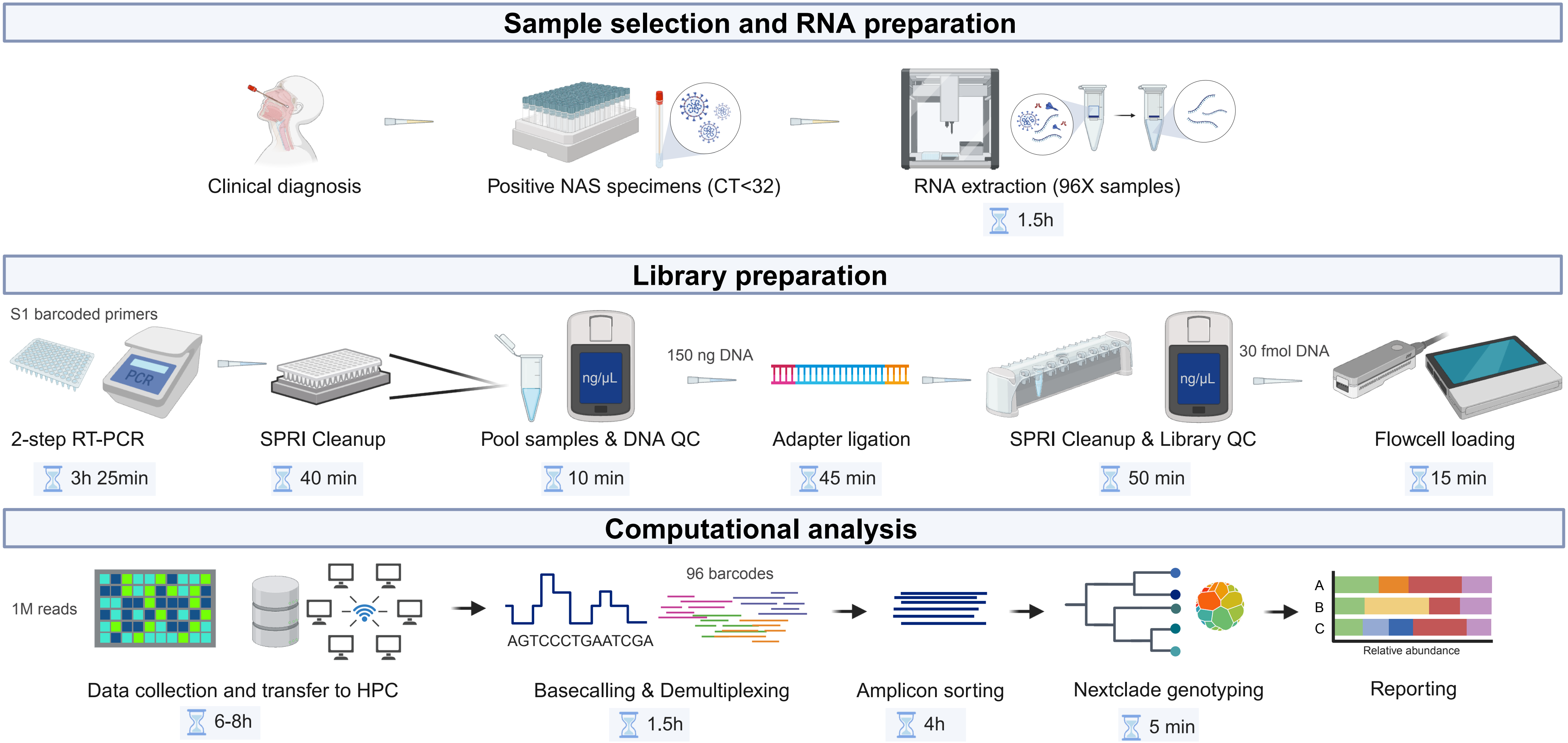

Supplementary Figure 1. SpikeID workflow for sample processing, library preparation and data analysis. A) Clinical respiratory specimens with Ct ≤ 32 are processed for automated RNA extraction. B) Sequencing libraries are prepared with the Oxford Nanopore Technologies (ONT) ligation sequencing kit (SQK-LSK109) with native barcodes (EXP-NBD196); and sequenced on a flowcell to up to 1M reads. C) Analysis is performed with custom in-house computational pipeline that includes high accuracy basecalling. Consensus sequences are assembled de-novo by a custom assembly pipeline and genotyped with Nexclade. Figure created with BioRender.com. Created in BioRender.
Van bakel, H. (2025) https://BioRender.com/p30h622

Supplementary Figure 2. Direct acyclic graph of the SpikeID analysis pipeline, from data acquisition to genotyping.

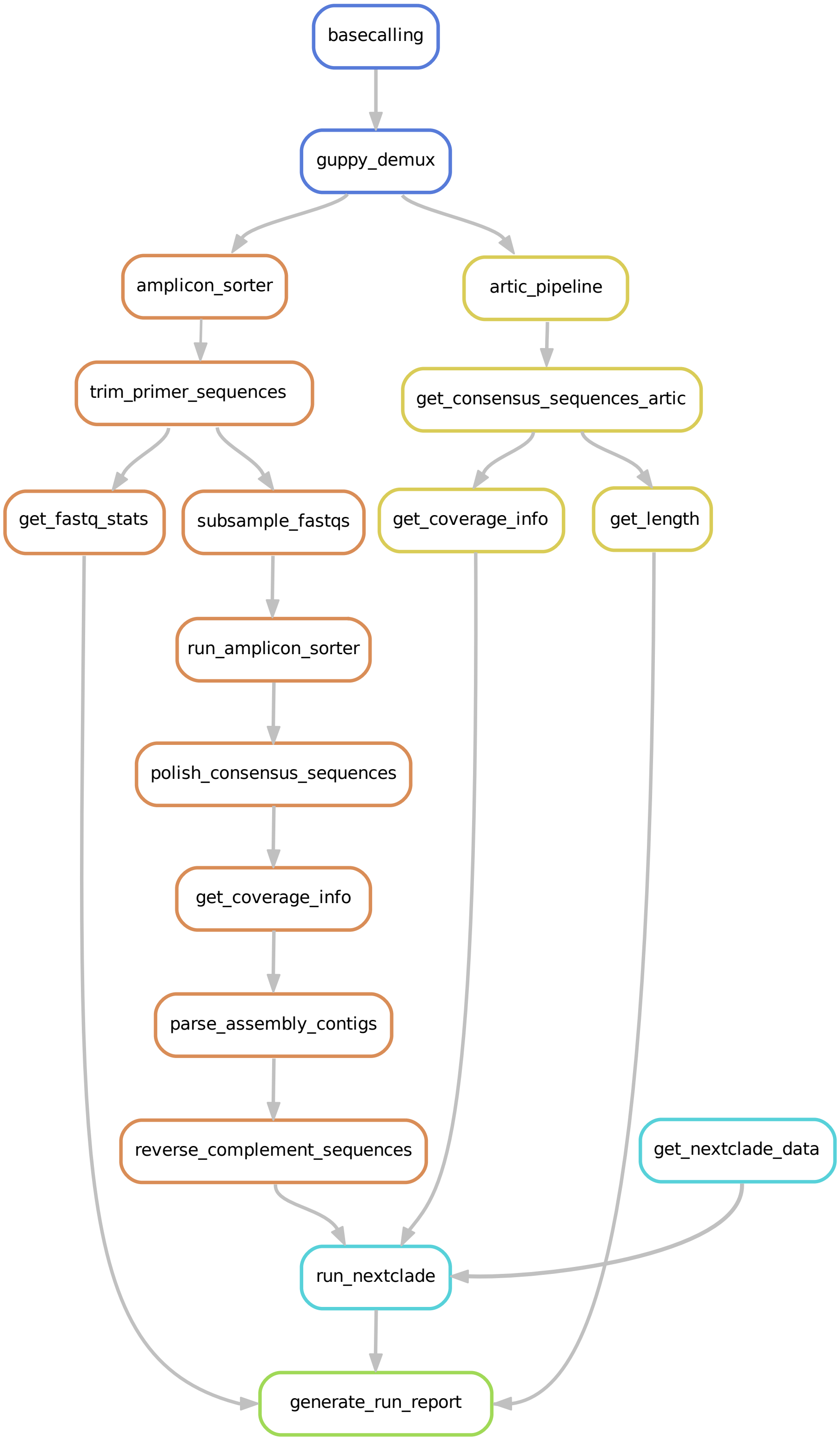

Supplementary Figure 3. Algorithm for lineage aggregation based on prevalence threshold.

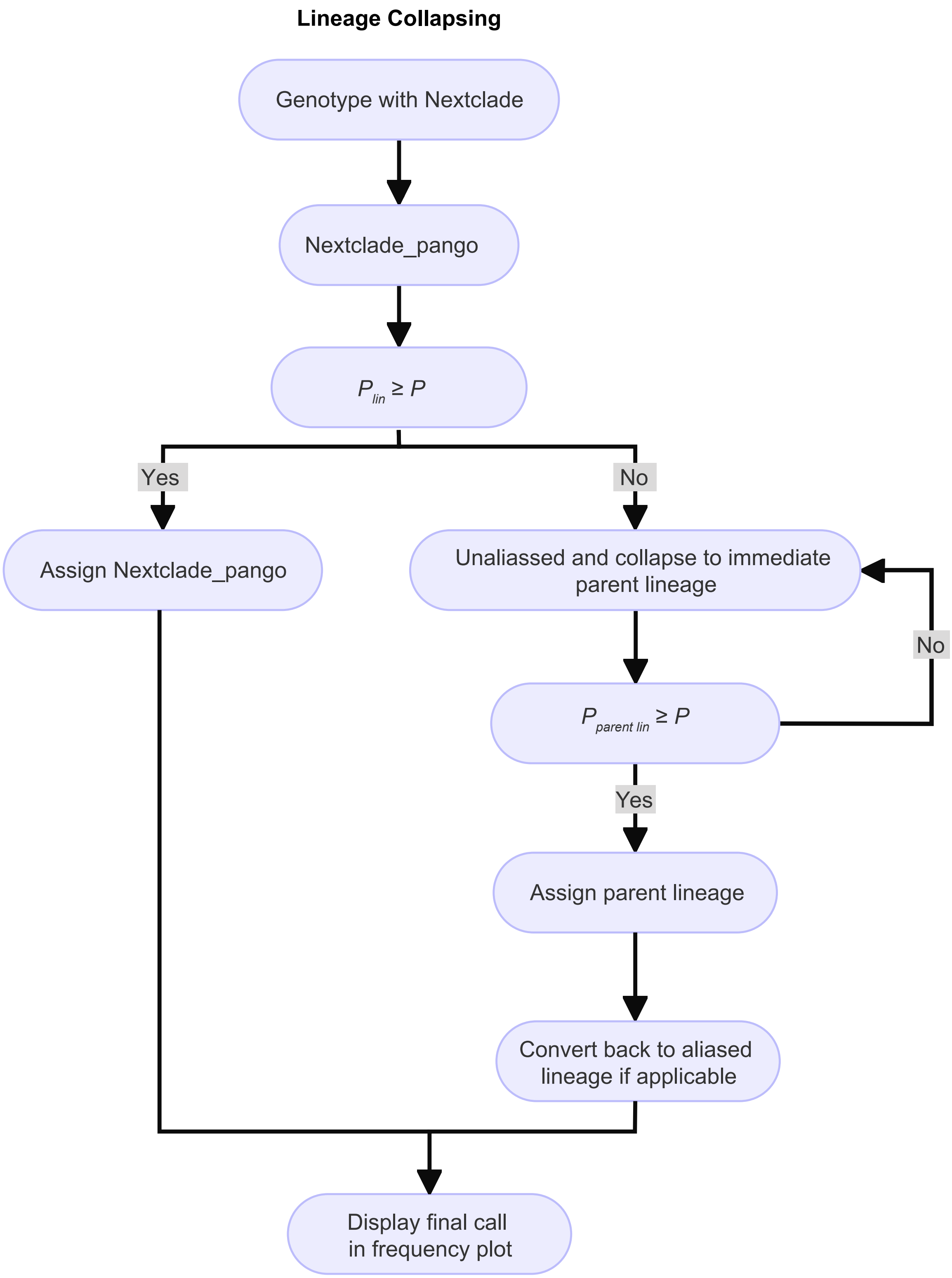

49 Spadina Ave. Suite 200
Toronto ON M5V 2J1 Canada
www.biorender.com

Confirmation of Publication and Licensing Rights - Open Access

July 25th, 2025

Subscription Type:	*Institution - Academic*
Agreement number:	*UN28JXBESO*
Publisher Name:	*Journal of Clinical Virology*
Figure Title:	*Supplementary Figure 1. SpikeID workflow for sample processing, library preparation and data analysis.*
Citation to Use:	*Created in BioRender. Van bakel, H. (2025) https://BioRender.com/p30h622*

To whom this may concern,

This document ("Confirmation") hereby confirms that Science Suite Inc. dba BioRender ("BioRender") has granted the following BioRender user: Ana Gonzalez-reiche ("User") a BioRender Academic Publication License in accordance with BioRender's Terms of Service and Academic License Terms ("License Terms") to permit such User to do the following on the condition that all requirements in this Confirmation are met:

1) publish their Completed Graphics created in the BioRender Services containing both User Content and BioRender Content (as both are defined in the License Terms) in publications (journals, textbooks, websites, etc.); and
2) sublicense such Completed Graphics under "open access" publication sublicensing models such as CC-BY 4.0 and more restrictive models, so long as the conditions set forth herein are fully met.

Requirements of User:

1) All Completed Graphics to be published in any publication (journals, textbooks, websites, etc.) must be accompanied by the following citation either as a caption, footnote or reference for each figure that includes a Completed Graphic:
"Created in BioRender. Van bakel, H. (2025) https://BioRender.com/p30h622 ".
2) All terms of the License Terms including all Prohibited Uses are fully complied with. E.g. For Academic License Users, no commercial uses (beyond publication in journals, textbooks or websites) are permitted without obtaining or switching to a BioRender Industry Plan.
3) A Reader (defined below) may request that the User allow their figure to be a public template for Readers to view, copy, and modify the figure. It is up to the User to determine what level of access to grant.

Open-Access Journal Readers:

Open-Access journal readers ("Reader") who wish to view and/or re-use a particular Completed Graphic in an Open-Access journal subject to CC-BY sublicensing may do so by clicking on the URL link in the

applicable citation for the subject Completed Graphic.

The re-use/modification options below are available after the Reader requests the User to adapt their figure as a BioRender template and the User has granted such access.

1) View-Only/Free Plan Use: A Reader who wishes to only view the Completed Graphic may do so in the BioRender Services as either a BioRender Free Plan user or simply as a viewer. By becoming a BioRender Free Plan user, the Reader may view, modify and re-use the Completed Graphic as permitted under BioRender's [Basic License Terms](#) (e.g. personal use only, no publishing or commercial use permitted).
2) Re-Use/Publish with No Modifications: For any re-use and re-publication of a Completed Graphic with no modification(s) to the Completed Graphic made by the Reader, a Reader may do so by citing the original author using the citation noted above with the Completed Graphic. The Reader must also comply with the underlying License Terms which apply to the Completed Graphic as noted above (e.g. no commercial use for Academic License).
3) Re-Use/Publish with Modifications: For any re-use and re-publication of a Completed Graphic with a modification(s) made by the Reader, the Reader may do so by becoming a BioRender user themselves under either an Academic or Industry Plan, citing the original author using the citation noted above with the Completed Graphic and complying with the applicable License Terms.

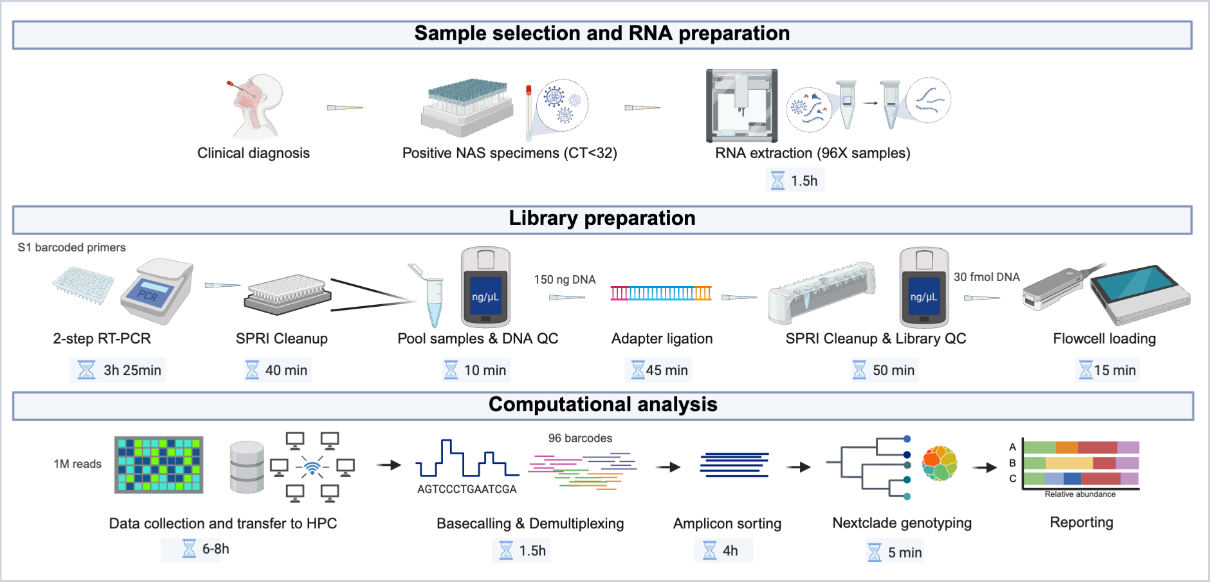

For any questions regarding this document, or other questions about publishing with BioRender, please refer to our [BioRender Publication Guide](#), or contact BioRender Support at support@biorender.com.
